# Supplementary figures and images for: Measuring Interprofessional Collaboration’s Impact on Healthcare Services Using the Quadruple Aim Framework: A Protocol Paper
Source: Int J Environ Res Public Health. 2023 May 1;20(9):5704. doi: 10.3390/ijerph20095704 (PMC10178681; doi:10.3390/ijerph20095704)

Supplementary Material

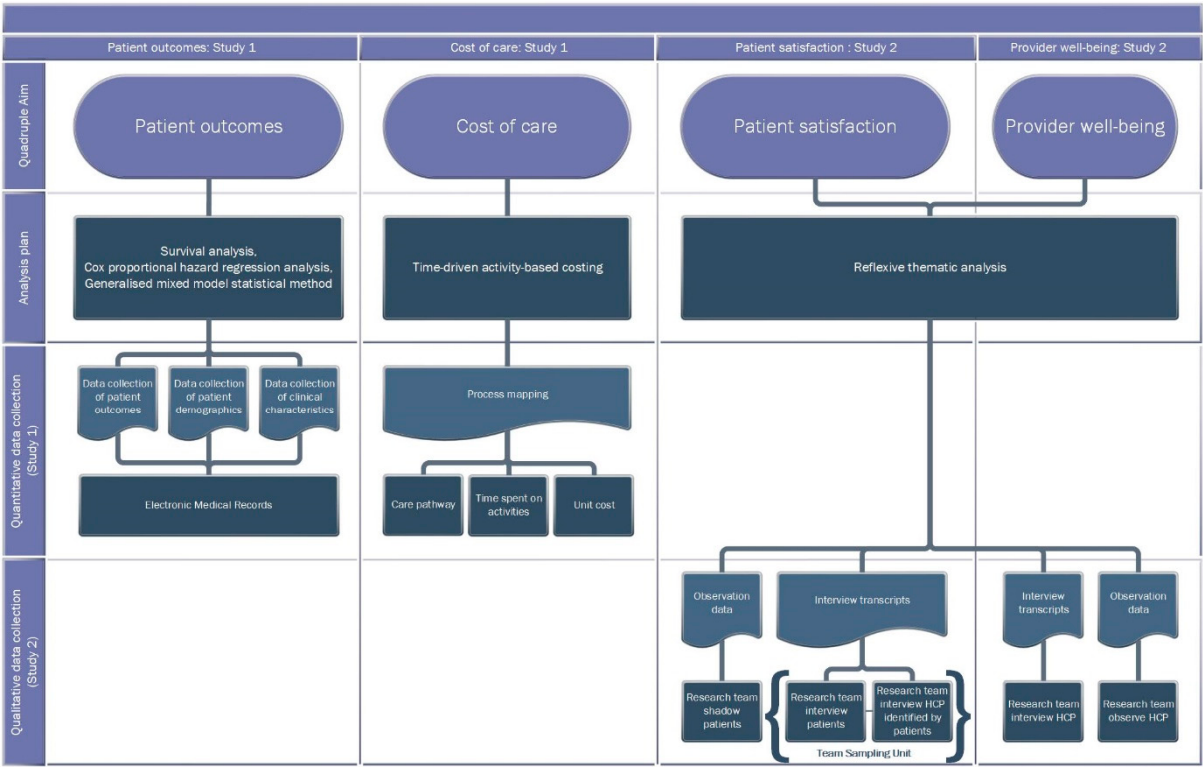

Figure S1. Protocol Workplan.

Supplement: Supplementary file 1 [file ijerph-20-05704-s001.zip › ijerph-2249127-supplementary.pdf]
